# SARS-CoV-2 and Environmental Changes: The Perfect Storm

**DOI:** 10.3390/cimb46110703

**Published:** 2024-10-23

**Authors:** Mario Caldarelli, Pierluigi Rio, Vincenzo Giambra, Ivana Palucci, Antonio Gasbarrini, Giovanni Gambassi, Rossella Cianci

**Affiliations:** 1 Department of Translational Medicine and Surgery, Catholic University of Sacred Heart, 00168 Rome, Italy; mario.caldarelli01@icatt.it (M.C.); pierluigi.rio01@icatt.it (P.R.); antonio.gasbarrini@unicatt.it (A.G.); giovanni.gambassi@unicatt.it (G.G.); 2 Fondazione Policlinico Universitario A. Gemelli, Istituto di Ricerca e Cura a Carattere Scientifico (IRCCS), 00168 Rome, Italy; ivana.palucci@unicatt.it; 3 Institute for Stem Cell Biology, Regenerative Medicine and Innovative Therapies (ISBReMIT), Fondazione IRCCS “Casa Sollievo della Sofferenza”, 71013 San Giovanni Rotondo, Italy; v.giambra@operapadrepio.it; 4 Dipartimento di Scienze Biotecnologiche di Base, Cliniche Intensivologiche e Perioperatorie-Sezione di Microbiologia, Catholic University of Sacred Heart, 00168 Rome, Italy

**Keywords:** COVID-19, climate change, JN.1

## Abstract

The COVID-19 pandemic has had a significant impact on the global economy. It also provided insights into how the looming global climate crisis might be addressed, as there are several similarities between the challenges proposed by COVID-19 and those expected from the coming climate emergency. COVID-19 is an immediate health threat, but climate change represents a more gradual and insidious risk that will lead to long-term consequences for human health. Research shows that climate change, air pollution and the pandemics have a negative impact on health. Recent studies show that COVID-19 mortality increases with climate extremes. The goal of our review is to analyze the clinical findings of COVID-19 and how they are affected by the climate change, while also providing insight into the emergence of new variants and their ability to evade the immune system. We selected and synthesized data from primary studies, reviews, meta-analyses, and systematic reviews. Selection was based on rigorous methodological and relevance criteria. Indeed, a new variant of SARS-CoV-2, named JN.1, has emerged as the dominant, first in the United States and then worldwide; the variant has specific mutations in its spike proteins that increase its transmissibility. According to the World Health Organization (WHO), JN.1 is currently the most reported variant of interest (VOI), having been identified in 132 countries. We highlight the link between climate change and pandemics, emphasizing the need for global action, targeted medical approaches and scientific innovation.

## 1. Introduction

The global COVID-19 pandemic landscape continues to evolve due to the emergence of new strains of the SARS-CoV-2 virus. The World Health Organization (WHO) recently classified a new strain, JN.1, as a “variant of interest” (VOI), underscoring its importance in the ongoing fight against the virus [1]. Moreover, the Centers for Disease Control and Prevention (CDC) in the United States is implementing a comprehensive surveillance plan to monitor the emergence and spread of these variants, exemplifying a collaborative global approach.

The JN.1 variant harbors several mutations within the spike gene, making it difficult to understand the behavior of the virus. This discovery has led to an enhanced surveillance program that includes genomic analysis, wastewater monitoring, traveler tracking, and digital public health surveillance in combination with existing public health systems [2].

Saleh et al. found a strong positive association between exposure to PM^2.5^ and PM^10^ and the development of long COVID. Their analysis also showed a significant association between air pollution and shortness of breath, especially when associated with PM^2.5^ exposure [3].

The purpose of our review is to analyze how climate change acts synergistically with SARS-CoV2 and to discuss how this association determines a more severe clinical scenario.

We also wanted to provide an update on new variants of interest that are emerging and how they might be able to evade the immune system.

Finally, we provide possible insights into new prevention and treatment strategies.

We performed a comprehensive search of electronic databases such as PubMed, MEDLINE, Embase, and Google Scholar, using keywords such as “SARS-CoV-2 new variants”, “SARS-CoV-2 JN1”, “immunological escape”, and “climate change”. We included original and review articles written in English and published in peer-reviewed journals between 1 January 2020 and 2024. Selection was based on relevance, study design, methodology, and included both small and large studies as well as case reports. In addition, a manual search of references cited in the articles was conducted to expand the pool of studies to consider.

## 2. Climate Change and Diseases

Climate change is a pressing issue for modern medicine, as its impact on human health continues to grow.

The adverse effects of climate, including changes in sea level, heat waves, extreme weather phenomena and greenhouse gas emissions, affect the quality of food and water, economic security and the possibility of building houses and workplaces, particularly in underdeveloped countries that cannot appropriately face these hazards [4].

According to the 2024 Europe report of the Lancet Countdown on health and climate change, “climate change is not a future threat, but a present reality with deadly consequences”. The authors focused on the negative impacts of climate change on human health, emphasizing the ultimate outcomes, exposures, and vulnerabilities [5].

**Figure 1:**
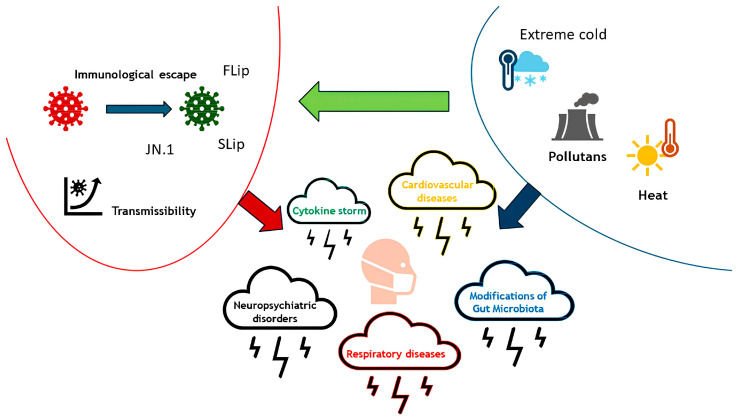
The figure summarizes how climate change (pollution, particulate matter and extreme temperatures) affects the transmissibility and selection of new variants of SARS-CoV-2. In addition, the synergy between SARS-CoV-2 and climate change leads to several diseas.es.

Using data from two large-scale European surveys, Stefkovics et al. found that concern about climate change increased between 2016 and 2021, particularly during the COVID-19 pandemic [6]. Indeed, climate imbalance has contributed to the outbreak of SARS-CoV-2 infection, due to temperature and humidity changes promoting viral survival, environmental pollution and climate-related changes in vegetation, nutrition and human activities, enhancing viral transmission and influencing clinical presentation, as summarized in Figure 1 [7].

According to Weaver et al., the environment can influence COVID-19 through four mechanisms, including pre-existing health conditions that are affected by the environment and can worsen disease severity; an imbalance in the immune system; survival and transmission of the virus; and behavioral habits that can increase exposure to the virus [8].

Research has shown that global warming and other climate events can lead to poorer health outcomes and increased mortality, particularly among vulnerable populations such as the elderly and those with chronic diseases [9,10].

For instance, extreme heat significantly impacts cardiovascular health, increasing the hospitalizations for both cardiac and cerebrovascular events.

Patients with respiratory diseases such as asthma, chronic obstructive pulmonary disease (COPD), pulmonary fibrosis and lung cancer are more vulnerable to the adverse effects of high temperatures, extreme weather, air pollutants, allergens and microbial exposure; In fact, an increased occurrence of disease exacerbations, hospitalizations and deaths has been reported due to climate change. Moreover, environmental exposures may trigger allergies, new-onset asthma, COPD and lung cancer [11,12].

For instance, higher levels of carbon dioxide (CO_2_) in the environment lead to increased pollen production by plants. In addition, extreme rainfall and flooding can promote mold growth. Both pollen and mold allergens contribute to the release of inflammatory and immunomodulatory mediators, resulting in sensitization and allergies [13].

Among the main drivers of heat-dependent respiratory exacerbations, PM and ozone play a pivotal role, as well as living in large urban contexts affected by air pollution and heatwaves, and those lacking green spaces [14]. A Swedish case-crossover study showed that household exposure to PM and dust was associated with a higher risk of positive SARS-CoV-2 PCR tests in young adults, supporting the association between environmental pollution and COVID-19 [15].

Thi Khanh et al. found that cold temperatures, especially in combination with moderate to high air pollution, may increase the risk of SARS-CoV-2 infection. On the other hand, exposure to air pollutants during infection combined with high temperatures may lead to post-COVID-19 conditions [16].

A study evaluating the impact of indoor temperature on patients with COPD found that heat has a detrimental effect on respiratory symptoms, such as cough and dyspnea, which further worsen in the presence of high concentrations of indoor fine PM and nitrogen dioxide [17]. A Korean case-crossover study by Lee and Yon examined the relationship between ambient temperatures and emergency department (ED) visits. Their results showed that both hot and cold temperatures are associated with an increased risk of ED visits. Hot temperatures are associated with an increased risk of influenza, pneumonia, and upper respiratory tract infections; on the other hand, cold temperatures are associated with an increased risk of all respiratory diseases [18].

## 3. SARS-CoV-2 and Climate Change

COVID-19 is an example of an emerging infectious disease whose spread from birds or bats to other animals and humans has been facilitated by changing weather patterns, such as extreme heat and rainfall [19]. The warmth of sunlight is vital to humans, as many organisms depend on it for survival. Conversely, reduced exposure to sunlight often correlates with an increased spread of disease-carrying viruses [20].

Historically, disease outbreaks that became epidemics and pandemics have been linked to atmospheric changes. Pandemic diseases, such as the 1918 flu, the Asian flu (1956–1958), the Hong Kong flu (1968), and more recently COVID-19 (2019), resulted in more than one million deaths and occurred primarily during winter months or periods of low temperatures [21].

Recently, there has been an increase in the number of studies examining the relationship between environmental factors, such as temperature and humidity, and coronavirus viability, transmission, and survival [22].

According to Diao et al., temperature is more important in transmitting SARS-CoV-2 and allowing it to persist on surfaces [23]. Research has consistently shown that several meteorological factors, including relative humidity, precipitation, temperature, and wind speed, significantly influenced the dynamic response of the pandemic, either facilitating or impeding the spread of the novel coronavirus [24].

Lagtayi et al. highlighted the critical role of temperature in disease transmission, citing higher transmission rates of MERS-CoV in African countries with warm and dry climates. Similarly, the highest number of COVID-19 cases was observed in temperate regions with dry climates, particularly in Western Europe, China, and the United States, while countries closer to the equator reported fewer cases [21].

Atmospheric instability, characterized by turbulent events, results in higher wind speeds and can hinder the dispersion of PM^2.5^ and PM^10^ in the environment [20]. A study conducted by Martins et al. during the COVID-19 outbreak found that regions with low wind movement had a higher correlation coefficient and exceeded the safe thresholds for PM^2.5^ and PM^10^. Consequently, these areas had a higher number of SARS-CoV-2 infections [25].

The potential for climate change to differentially affect SARS-CoV-2 transmission at different times has significant implications for understanding the dynamics of the selective spread of new variants. However, our understanding of how different viral variants respond to environmental factors remains limited. Smith et al., evaluating three variants (Alpha, Delta and Omicron BA.1) throughout England, have recently found that colder temperatures enhance the transmission of the Alpha variant, with no clear data about Delta and Omicron. The influence of climate is non-linear, since the impact of temperature on transmission rate is higher at lower temperatures [26].

Rendana and Idris had previously identified a relationship between the B.1.1.7 (Alpha) variant and meteorological factors (temperature, rainfall, and sunshine duration) that can influence the spread of the virus [27].

For emerging variants, colder temperatures may facilitate the faster transmission of a more contagious variant, allowing it to spread more rapidly through the population. Notably, while Delta is generally considered to have a higher intrinsic transmission rate than Alpha, Alpha demonstrated a higher transmission rate during its winter spread in England compared to Delta during spring and summer [28].

Smith et al. found no significant effect of climate during the Delta and Omicron waves, but this may be due in part to their ability to account for non-climate variations [26]. The enhanced ability of the Delta strain to evade the immune system may account for its superior transmission capabilities compared to other strains [29] Omicron quickly overtook Delta to become the dominant circulating strain [30] and that may also be explained by its ability to evade vaccine-induced immunity [31].

Temperature may also affect the in traction between SARS-CoV-2 and human ACE2 by affecting the conformation of the spike glycoprotein [32]. According to Gong et al., low temperatures increase the affinity and binding of SARS-CoV-2 Omicron subvariant spikes to the ACE2 receptor under laboratory conditions. Further research is needed to understand how this enhanced Spike-ACE2 interaction at colder temperatures might facilitate virus transmission in living organisms [33].

A significant number of people recently diagnosed with COVID-19 report a sore throat as their first symptom, often followed by congestion. Symptoms such as dry cough and a decreased sense of smell or taste, which were once common, are now reported less frequently [1].

Chong et al. conducted a retrospective population-based cohort study of all Singaporeans aged 18 years and older who were vaccinated during the COVID-19 wave, which was predominantly characterized by the JN.1 variant, from November 2023 to January 2024 [34]. The study used multivariable Cox regression analysis to evaluate the risk of SARS-CoV-2 infection and COVID-19-related emergency department visits or hospitalizations. The results were stratified by vaccination status and prior infection. Individuals who had received their last vaccination more than one year ago served as the reference group for comparison. During the JN.1 outbreak, a total of 28,160 SARS-CoV-2 infections were reported, resulting in 2926 hospitalizations and 3747 emergency department visits. Compared with individuals who received their last booster with ancestral monovalent vaccines at least one year ago, those who received an updated XBB.1.5 booster within 8 to 120 days had a lower risk of JN.1 infection. In addition, a reduced risk of COVID-19 hospitalization during the JN.1 outbreak was observed after the administration of the updated XBB.1.5 booster within the same timeframe, even among individuals with previous infections [34].

### 3.1. Cardiovascular System

Coronavirus outbreaks, such as Severe Acute Respiratory Syndrome (SARS) and Middle East Respiratory Syndrome (MERS), have been associated with significant increases in cardiovascular disease [35]. Cardiovascular complications, such as hypotension, myocarditis, arrhythmias, and sudden cardiac death, were common with SARS [35].

COVID-19 appears to have cardiac effects that are like those seen in previous outbreaks caused by other coronaviruses. Studies have shown that individuals with active COVID-19 may have an increased risk of acute myocardial infarction (MI) and ischemic stroke [36].

COVID-19 has the potential to trigger acute coronary syndromes (ACSs) through mechanisms such as plaque destabilization and an imbalance between supply and demand [37].

Kesici et al. have documented acute myocarditis occurring during symptomatic COVID-19 [38]. However, the exact incidence remains uncertain. Luetkens et al. conducted a prospective study in Germany of 100 patients who had recovered from COVID-19, including cardiac magnetic resonance (CMR) imaging [39].

Studies have shown that 60% of COVID-19 patients have persistent myocardial inflammation. Evidence of acute cardiac injury, as indicated by elevated troponin levels, has been observed in COVID-19 patients several days after the onset of fever, suggesting a link between myocardial damage and viral infection [40]. The mechanisms underlying SARS-CoV-2-induced myocardial injury remain unclear, but may involve the direct effects of SARS-CoV-2 on cardiac myocytes or the upregulation of ACE2 in the heart and coronary vasculature [41].

Cardiac injury can trigger the activation of the innate immune response, leading to the release of pro-inflammatory cytokines. In addition, it can activate adaptive autoimmune mechanisms through molecular mimicry. For example, cluster of differentiation 209 (CD209) is a receptor that may facilitate SARS-CoV-2 entry into immune cells within the heart and blood vessels [42]. CD209 is found on macrophages [43].

It is noteworthy that severe cases of COVID-19 are characterized by a systemic increase in cytokine levels, including interleukin (IL)-6, IL-2, IL-7, granulocyte colony-stimulating factor, C-X-C motif chemokine ligand 10, chemokine (C-C motif) ligand 2, and tumor necrosis factor alpha [44].

These findings are consistent with the cytokine-releasing syndrome (CRS) characteristics of COVID-19 [45].

Despite limited information on the prevalence of malignant arrhythmias in COVID-19 patients, including ventricular tachycardia (VT) and atrial fibrillation (AF), small clinical studies estimate that the incidence of new-onset AF in COVID-19 patients ranges from 3.6% to 6.7% [46].

Heart failure (HF) decompensation was the most common complication associated with COVID-19 through mechanisms such as direct viral infiltration, inflammation, or cardiac fibrosis [47]. The increased metabolic burden of COVID-19 may also reveal a previously undiagnosed heart failure or worsen existing ones [47].

Increased serum B-type natriuretic peptide (BNP) levels have been associated with a significantly increased risk of death. A meta-analysis showed that heart failure developed as a complication in 11.5% of COVID-19 patients [48].

In addition, cardiac myocytes contain a high density of ACE2 receptors, making these cells particularly susceptible to binding, entry and infection by SARS-CoV-2 [49]. The virus damages the cells, causing disruptions and dysfunctions that can eventually lead to damage to the heart muscle and a subsequent inflammatory response [50]. Endothelial cell damage has been implicated in the mechanisms underlying COVID-19. Endothelial cells in the heart and blood vessels are susceptible to cardiovascular and systemic complications. Some studies suggest that ACE2 receptors are found on endothelial cells and may directly facilitate infection, similar to their role in cardiac myocytes [51].

It should not be overlooked that cardiovascular disease is considered part of long COVID.

In a study of 153,760 patients, Xie et al. found that individuals with long COVID had a 1.6-fold increased risk of new-onset cardiovascular disease [52]. This includes arrhythmias, both non-ischemic and ischemic cardiomyopathies, as well as cerebrovascular and thrombotic disorders. Ayoubkhani et al. conducted another study of 47,780 patients and found that major adverse cardiovascular events were more than 1.5 times more common in patients with Long COVID than in control groups [53].

The association between SARS-CoV-2 infection, increased PM^2.5^ exposure, and higher mortality from ischemic heart disease suggests a possible interaction between infectious agents and air pollution in the development of atherosclerosis and ischemic heart disease [54].

The relationship among SARS-CoV-2 binding to cardiac pericytes and endothelial cells, endothelial dysfunction, microvascular damage, PM^2.5^ exposure, atherosclerosis, and myocardial ischemia is complex and multifaceted [55]. PM^2.5^ exposure has been associated with increased ACE2 expression in endothelial cells, which may increase the likelihood of SARS-CoV-2 infection. This can lead to a higher viral load and a stronger systemic response with inflammation and blood coagulation, which can increase the risk of acute coronary syndrome [56].

COVID-19 may affect the cardiovascular system because of an excessive inflammatory response caused by a disrupted immune system after viral infection. This response may be exacerbated when people are also exposed to PM^2.5^ [57].

This can lead to excessive release of cytokines (a condition called cytokine storm), activation of the inflammasome, and a pro-inflammatory environment in the blood vessels, with a subsequent diffuse intravascular coagulation and an increased risk of ischemic coronary events, such as plaque instability and coronary artery blockage by blood clots [58].

In a systematic review about climate change and cardiovascular health, Kazi et al. found that environmental stressors, such as extreme temperature and weather events (e.g., hurricanes), may increase cardiovascular morbidity and mortality, depending on the duration of the exposure [59]. However, in an early pandemic study, Benedetti et al. highlighted the existence of an inverse correlation between average monthly high temperatures and COVID-19-related deaths, suggesting that social distancing measures are more efficient in warmer months [60]. Heat stress can trigger thermoregulatory mechanisms, such as sweating, vasodilation, and increased blood flow to the skin. This activates the sympathetic nervous system, which increases heart rate and cardiac output. Dehydration from sweating can also lead to blood clots. This can damage heart function, especially in older people and those with heart diseases, who are more likely to become dehydrated, with electrolyte imbalances, blood pressure changes, heart attacks, heart failure, and arrhythmias [61,62].

Heat stress has been shown to increase the production of pro-inflammatory cytokines such as interleukin-1 and interleukin-6, which can damage blood vessels [62].

People with heart failure who have problems with blood vessel function and reduced blood flow caused by nitric oxide may also have changes in skin blood flow. They are more likely to become dehydrated and have electrolyte imbalances because they are taking diuretics such as furosemide [63].

Ohashi et al. analyzed the mortality related to temperature-sensitive cardiovascular and respiratory diseases during COVID-19 pandemic, collecting data from three Japanese cities from 2010 to 2019. They suggested that the COVID-19 pandemic could have decreased the number of deaths due to changes in people’s behavior, such as the reduced exposure to extreme temperature and the increased indoor activities, as observed particularly in older and vulnerable individuals. However, from a long-term perspective, the sedentary lifestyle promoted by COVID-19 may negatively impact on cardiovascular and respiratory health [64].

Interestingly, in a recent study focusing on the relationship between climate change and cardiovascular healthcare, the authors described the significant contribution of telemedicine, embraced by physicians during the COVID-19 pandemic, on climate change itself, given the reduction of CO_2_ emissions depending on patient travel [65].

### 3.2. Gastrointestinal System

Another target organ of the new variants remains the gastrointestinal system.

The virus affects the intestine by interacting with ACE2, leading to an increase in inflammatory cytokines, and compromising the mucosal barrier. In severe cases, this inflammation can lead to ulcers in the esophagus, stomach and duodenum [66]. More commonly, it results in symptoms such as nausea, vomiting, abdominal pain, and diarrhea [66].

It has been shown that the virus can be present not only in the intestine, but also in the immune cells in the digestive system, and can have an effect on the gut microbiome (GM) [67]. Yokohama et al. examined the GM of COVID-19 patients and found a decrease in diversity in association with the Alpha, Delta, and Omicron variants [68]. While overall diversity was similar between variants, the Omicron variant showed some differences. Patients with the Alpha variant had higher levels of short-chain fatty-acid-producing bacteria, such as *Catenibacterium, Ruminococcus*, and *Eubacterium*, compared to patients with the Delta variant. In addition, *Oscillospirales, Faecalibacterium, Catenibacterium*, and *Subdoligranulum*, which produce butyric acid, were more abundant in patients with the Alpha variant than in patients with the Omicron one [68].

In addition, extreme heat induces liver damage, consisting of inflammation, vasoconstriction and centrilobular necrosis [69].

COVID-19 may also cause elevated levels of alanine aminotransferase, aspartate aminotransferase, and lactate dehydrogenase. It is not easy to understand how COVID-19 damages the liver because liver cells have few ACE2 receptors, which means the virus does not directly damage them [70].COVID-19 infection can damage the liver by increasing intestinal barrier leakage and tissue damage [71]. Elevated levels of calprotectin can often indicate changes in the intestinal barrier that may be associated with the growth of harmful bacteria such as Enterobacteriaceae and *Escherichia coli* [72]. This chain of events can trigger a widespread inflammatory response, potentially leading to conditions such as Systemic Inflammatory Response Syndrome (SIRS) [73]. Inflammatory substances such as granulocyte-macrophage colony-stimulating factor (GM-CSF) and IL-6 can activate CD8+ cytotoxic T cells, TNF-α, and cause cytokine storms that can lead to liver cell death [74]. Bacteria and their waste products can travel through the hepatic portal vein to the liver, where they interact with receptors on liver cells and Kupffer cells called TLRs and NLRs. This can trigger the production of more inflammatory cytokines [75]. Bacteria such as *Clostridium* and *Peptostreptococcus* can disrupt the nitrogen balance by producing excess ammonia, which can cause metabolic problems in liver cells and contribute to liver damage [76]. This cascade of events may contribute to elevated blood levels of bilirubin and liver enzymes, such as alanine aminotransferase and aspartate aminotransferase. The changes in the gut microbiome are summarized in Table 1.

Environmental changes alter the soil microbiota, which in turn affects the food and causes shifts in the GM [77]. There is growing evidence in the literature that climate change and environmental pollutants play a significant role in reducing both macro- and micro-diversity. The ‘biodiversity hypothesis’ suggests that a decline in biodiversity in natural environments negatively affects the composition and activity of the human microbiome [78]. Mattoo et al. have studied the influence of the soil microbiome on several physiological processes, including digestion, vitamin and mineral production, mental health and immune function [79]. For example, climate-related changes in crop quality can alter the composition of the human GM, leading to a decrease in Bacteroides and an increase in Proteobacteria, which are commonly found in malnutrition, painting a picture of dysbiosis [80].

These climate-related changes in the GM are closely linked to the activation of innate immune responses [78]. Climate change can activate innate immune cells, such as neutrophils, eosinophils, monocytes, basophils, and mast cells, in several ways.

For instance, as observed by Presbitero et al., even a mild increase in body temperature may impair immune responses and trigger inflammation [81], thus highlighting the importance of temperature for physiological functions. Additionally, extreme heat has been linked to DNA damage, oxidative stress and the malfunctioning of cell death pathways [78]. Furthermore, the prolonged exposure to environmental dusts has proved to activate dendritic cells and macrophages, induce the release of inflammatory cytokines and chemokines, as well as impair the activation of T helper cells. As a result, both particulate and non-particulate triggers can activate similar pathways and responses, often working with NF-κB-dependent pathways and stimulating the NLRP3 inflammasome, leading to acute inflammatory responses [78].

Extremely high temperature has been linked to GI damage in both animal models and humans, generally characterized by an increased intestinal permeability. Interestingly, short-term changes in environmental temperature may affect DNA methylation and alter epigenetics [70].The existence of a relationship between heatwaves, infectious gastroenteritis and flares of inflammatory bowel disease has been suggested.

We can hypothesize that the state of dysbiosis and chronic inflammation caused by both COVID-19 and the climate are mutually reinforcing and help to explain the increased incidence of functional and inflammatory gastrointestinal diseases. Although chronic exposure to PM and chemicals has been predominantly linked to the development of respiratory diseases, air pollution has also showed to impact on gastrointestinal health. On the one hand, poor air quality and PM increase the risk of COVID-19; on the other hand, pollution and COVID-19 synergically contribute to gastrointestinal disorders [82].

A healthy gastrointestinal system is also based on diet. Food systems are both a driver of climate change and environmental degradation, and will be significantly affected by climate change and environmental degradation [83]. Climate change affects the composition and nutrient availability of certain foods, resulting in changes in nutritional quality. For example, higher carbon dioxide levels can reduce the protein and mineral content of crops, affecting their nutritional value, yield and overall quality [84]. Climate-related changes in crop quality can alter the composition of the human GM, leading to a decrease in Bacteroides and an increase in Proteobacteria, which are commonly found in malnutrition, painting a picture of dysbiosis [83]. The interactions between SARS-CoV2 and host-specific cellular targets can trigger severe human metabolic reprogramming/dysregulation (HMRD), leading to the rewiring of sugar, amino acid, lipid and nucleotide metabolism [85]. This also results in altered or impaired bioenergetics, immune dysfunction and redox imbalances in the body. During the infection process, the virus hijacks two major human receptors, ACE-2 and/or neuropilin (NRP)-1, for initial attachment to the cell surface [86]. It then uses two key host proteases, TMPRSS2 and/or furin, to enter the cell, and finally uses an endosomal enzyme, cathepsin L (CTSL), for the fusogenic release of its viral genome [87].

In addition, the clinical manifestations of long COVID and associated metabolic dysfunction suggest the involvement of several pathobiological mechanisms [88].

Therefore, precision nutritional protocols aimed at addressing systemic impairments and “resetting” virus-induced HMRD represent the most relevant and effective strategy to combat the post-acute sequelae of SARS-CoV-2 [89].

It is therefore essential to protect the quality of our diet from climate change, as it acts as both a preventative measure and a treatment for COVID-19. In fact, a safe, balanced diet, rich in fruits and vegetables, which contribute to the establishment of a healthy varied GM, has been linked to a decreased risk and lower severity of COVID-19 [90].

### 3.3. Neurological and Psychiatric Disorders

COVID-19 may also damage the brain by affecting body systems, such as low oxygen or reduced blood flow.

Guerrero et al. reported the case of a 58-year-old man who developed neurological symptoms such as loss of smell, muscle spasms, fluctuating consciousness, rapid eye movements, and asymmetric muscle stiffness and slowness after a severe SARS-CoV-2 infection. Brain scans showed a decrease in dopamine uptake on both sides of the brain, with one side more affected than the other [89].

A 34-year-old woman was diagnosed with SARS-CoV-2-induced acute disseminated encephalomyelitis, an autoimmune disease of the central nervous system, as reported by Ghosh et al. [91]. The patient experienced a series of movement disorders, primarily muscle spasms, followed by brain dysfunction. She was eventually diagnosed with SARS-CoV-2-induced acute disseminated encephalomyelitis, which was successfully treated with intravenous methylprednisolone and intravenous immunoglobulin.

In the case of long COVID, neurological symptoms are particularly suggestive. They include loss of smell (anosmia), altered taste (dysgeusia), shortness of breath, chronic cough, severe fatigue, difficulty breathing (dyspnea), joint pain, chest pain, palpitations, orthostatic intolerance, memory and concentration problems often referred to as “brain fog”, and psychosocial challenges such as anxiety, depression, and insomnia [92].

Studies from around the world have consistently shown that fatigue is the most common and disabling symptom of long COVID, regardless of how severe the initial illness was or whether there were breathing problems [93].

While millions of people have reported memory problems related to COVID-19, we do not fully understand how these problems occur or how vaccines might help. Interleukin-1, a key component of the immune response to SARS-CoV-2, was found at higher levels in the hippocampus of COVID-19 patients. Vanderheiden et al. showed that the intranasal infection of C57BL/6J mice with the SARS-CoV-2 beta variant resulted in the migration of Ly6Chi monocytes into the central nervous system and the activation of microglia [94]. While H1N1 influenza does not increase brain IL-1β levels, SARS-CoV-2 does, leading to a sustained reduction in hippocampal neurogenesis and contributing to long-term cognitive deficits. Importantly, vaccination with a low dose of adenovirus-vectored spike protein can prevent hippocampal IL-1β production during breakthrough infection, preserving neurogenesis and preventing memory impairment [94].

The scoping review by Louis et al. found 364 articles that fell into three main themes, including extreme weather events and temperature changes, emerging neuroinfectious diseases, and the effects of pollutants [95]. The studies included in the review highlighted the association between worsening neurological symptoms and temperature variability, the increase in tick-borne infections due to a warming climate, and the impact of air pollutants on the incidence and severity of cerebrovascular disease. They found that both temperature extremes and variability were associated with increased incidence and severity of stroke, migraine headaches, hospitalizations in dementia patients, and exacerbations of multiple sclerosis.

As observed by Amiri et al., global warming is changing neurological practice due to its impact on several neurological diseases in terms of incidence, prevalence, mortality, and morbidity [96].

For example, extremely high temperatures increase the incidence and mortality for cerebrovascular events and impair the symptoms of neurodegenerative diseases [97].

At the brain level, heat exposure is responsible for mitochondrial membrane dysfunction, which increases the release of reactive oxygen species (ROS) and cytochrome c (Cyt c), ultimately promoting the mechanisms of apoptosis and therefore neurodegeneration. In addition, high temperatures lead to increased glutamate levels and increased intracellular calcium levels, which are associated with excitotoxicity and seizures [98].

Similarly, exposure to air pollutants, particularly PM^2.5^ and nitrates, has been associated with increased risk of stroke, more severe headaches, increased risk of dementia, and exacerbation of Parkinson’s disease and multiple sclerosis [95].

From this, it is evident that there is a synergy between climate change, pollutants, and the wide range of neurological manifestations that can be caused by SARS-CoV-2.

## 4. Emergent Variants of SARS-CoV-2: Mutations and Immunological Escape

Climate change may have contributed to the emergence and transmission of SARS-CoV-2, and probably also to some of the clinical consequences of infection [7]. Viruses constantly replicate, mutate and evolve within individuals. This evolutionary process not only allows them to adapt to their hosts, but also equips them for efficient transmission among humans [99]. As viral lineages diverge into distinct strains, the dynamics become more complex, with competition between lineages and the potential for some to go extinct [100]. These dynamics influence the emergence and persistence of specific variants and shape the trajectory and evolution of the virus [101].

The extraordinary recent climate changes, such as the confirmation of 2023 as the hottest calendar year in global temperature records going back to 1850 [102], have clearly influenced the emergence of new SARS-CoV-2 variants that require attention.

The SARS-CoV-2 BA.2.86 lineage, first detected in August 2023, is genetically distinct from the current Omicron XBB lineages, such as EG.5.1 and HK.3. Compared to XBB and BA.2, BA.2.86 has over 30 mutations in its spike protein, which may allow it to evade the immune system [103]. BA.2.86 has evolved into its progeny, JN.1 (BA.2.86.1.1), which emerged in late 2023 [104].

Yang and his colleagues investigated JN.1’s ability to evade the immune system using a pseudovirus-based neutralization assay from the plasma of individuals recovering from XBB infection [105]. JN.1 demonstrates a superior ability to evade the immune system compared to BA.2.86. [106].

JN.1 distinguishes itself from the predominant variant XBB.1.5 by harboring more than 30 spike protein mutations. Notably, with the advent of JN.1, certain variants have been identified possessing mutations at crucial sites within the spike protein, including S:L455F, S:F456L, and R346 [107].

The first to emerge were the so-called FLip variants, characterized by the S:L455F and F456L mutations within the XBB.1.5 backbone [108]. Both S:F456L and S:L455F significantly increase ACE2 affinity, and their combination has a synergistic effect [109]. In addition, S:L455F confers a degree of immune evasion [110].

The evolution of JN.1 continues, as evidenced by the emergence of ‘slip’ variants with the S456L mutation adjacent to L455S. JN.1 itself has the F456L mutation in the spike protein, and the ‘S’ in its name indicates the presence of the L455S mutation, which is also found in JN.1 [111]. Recently, the FLiRT variant has appeared with an additional R346T mutation in the SLip backbone. Another FLiRT variant, KP.2, has both the R346T and F456L mutations in S1 and a V1140L mutation in S2 [112].

The new mutations of interest are summarized in Table 2.

JN.1 showed significantly lower cell–cell fusion activity than D614G, similar to other Omicron variants [113]. The D614G mutation in SARS-CoV-2 is a change in the spike protein that replaces aspartic acid with glycine at position 614. Laboratory experiments have shown that this mutation increases infectivity [114]. A structural analysis suggests that the D614G mutation alters the shape of the spike protein, making it more likely to bind to ACE2 and enter cells [115].

An examination of the prevalence of 614D and 614G variants over time, based on data from global sequence databases, suggests that regions initially reporting 614D viruses during the early pandemic often had a higher frequency of 614G viruses later on [116]. Residue R346 within the receptor binding motif (RBM) forms critical interactions with residue D450 in the BA.2.86 line, potentially hindering receptor binding [117].

The R346T mutation removes this interaction, potentially increasing the binding affinity of ACE2. Conversely, mutations such as F456L and L455S in strains JN.1 and SLip reduce the hydrophobicity of the RBM, potentially leading to a decrease in ACE2 binding affinity [118].

## 5. Future Strategies

In recent years, humanity has faced significant challenges from infectious diseases. For example, diseases that were once under control have re-emerged, and pathogens have evolved into drug-resistant strains [119]. Similarly, for COVID-19 a new variant JN.1 has become dominant and scientists are studying the characteristics and its potential impact. While existing antibodies offer some protection, JN.1’s unique mutations and rapid transmission pose significant challenges to global COVID-19 efforts [1].

In this review, we have considered the potential influence that climate change can have on the onset, transmission, and health consequences of the COVID-19 pandemic. We have presented evidence suggesting that climate change may have played a role in the emergence and spread of new SARS-CoV-2 variants. Changes in temperature and humidity favor the survival of viruses, while the effects of industrial pollution led to increased coughing and sneezing, releasing highly infectious aerosols into the air. Together, these three factors create a scenario that is more conducive to the spread of infection [120]. We propose that climate change contributes to conditions that favor the development of more severe disease symptoms.

Climate change has caused certain communities to change their dietary habits, affecting both the quantity and quality of the food they consume. These changes often manifest themselves through changes in the GM, which subsequently influence the types of immune responses [120].

It is fascinating to observe the common role of IL-1 in all these processes.

Attur et al. examined the effects of single nucleotide variants (SNVs) in the IL1RN gene, which encodes the anti-inflammatory interleukin-1 receptor antagonist (IL-1Ra), on cytokine release syndrome (CRS) and mortality in patients with acute SARS-CoV-2 infection [121].

The IL1RN haplotype CTA and the C/C variant of rs419598 were associated with reduced CRS and mortality in men with acute SARS-CoV-2 infection, according to research by Attur et al. This suggests that the IL1RN pathway plays a role in determining the severity of COVID-19 by influencing natural anti-inflammatory mechanisms [121].

Investigating IL-1 inhibitors as a potential precision therapy is probably a viable future avenue.

Studying new mutations also remains essential to develop updated and effective vaccines.

In a recent study, Soudani et al. compared how well the updated XBB.1.5, the original Prototype Wuhan-1, and the bivalent Prototype + BA.5 vaccines worked against the EG.5.1 Omicron variant of SARS-CoV-2 in hamsters [122]. They found that both the XBB.1.5 and bivalent vaccines induced serum-neutralizing antibodies against EG.5.1, while the Prototype did not [122].

A One Health approach that recognizes the interconnection of human, animal and environmental health is needed to address these complex challenges.

It is essential to apply precision medicine and adopt a holistic perspective, and the concept of the “exposome” is becoming increasingly important in this context.

The exposome is the term for all the internal and external influences that individuals experience throughout their lives, including biological, chemical, physical, psychological, social, and economic factors. [123]. It looks at the duration and intensity of these exposures and how they interact over time. This approach combines several fields of science, particularly toxicology, to understand the long-term health effects of toxic exposures [123].

These preventive and therapeutic strategies, which consider these multiple interactions, can reduce the risk of incidence and morbidity of new SARS-CoV-2 variants while promoting sustainable and precise global health.

## 6. Conclusions

A thorough analysis of the various climate risks that affect COVID-19 transmission, lived experience, and responses is needed. Implementing new interventions that emphasize long-term projects and outcomes, along with global collaboration, will help address both climate change and the risk of future pandemics.

Thus, an integrated approach would be critical to improve our understanding of the environmental factors that enhance COVID-19 transmissibility and increase morbidity. This approach would allow for the development of targeted therapies to address the environmental drivers of this disease.

In addition, increasing the distribution and production of updated vaccines will be a key strategy to protect people from the risk of new variants. As more people are vaccinated, the expectation is that virus circulation will decrease, resulting in fewer mutations.

Finally, the investigation on IL-1 inhibitors and drugs capable of inhibiting inflammasome activity are likely to be a promising avenue for the future.

## Figures and Tables

**Table 1:** Changes in the gut microbiome observed in patients infected with emerging variants.

GM of COVID-19 Patients	Changes Observed	Reference
α-diversity	decreased	[69]
β diversity	no significant differences	
short-chain fatty-acid-producing bacteria (*Catenibacterium*, *Ruminococcus*, and *Eubacterium)*	more abundant in patients infected with the Alpha strain compared to the Delta	[69]
species that generate high levels of ammonia (*Clostridium* and *Peptostreptococcus)*	increased	[76]

**Table 2:** Summary of emerging variants with their respective mutations of interest.

SARS-CoV-2 Emergent Variants	Mutation of Interest	Reference
JN.1 (BA.2.86.1.1)	more than 30 spike protein mutations	[106]
FLip	S:L455F and F456L	[108]
SLip	S456L mutation adjacent to L455S	[112]
FLiRT	R346T mutation in the backbone of SLip	[111]
KP.2	R346T and F456L mutations	[112]

## Data Availability

Not applicable.
